# Granulocyte colony-stimulating factor-producing lung cancer complicated with antiphospholipid antibody syndrome: a case report

**DOI:** 10.1093/jscr/rjae361

**Published:** 2024-05-30

**Authors:** Ryusei Yoshino, Masaki Nakatsubo, Nanami Ujiie, Sayaka Yuzawa, Kensuke Ishida, Masahiro Kitada

**Affiliations:** Department of Thoracic Surgery and Breast Surgery, Asahikawa Medical University Hospital, 2-1-1-1 Midorigaoka Higashi, Asahikawa, Hokkaido 078-8510, Japan; Department of Thoracic Surgery and Breast Surgery, Asahikawa Medical University Hospital, 2-1-1-1 Midorigaoka Higashi, Asahikawa, Hokkaido 078-8510, Japan; Department of Thoracic Surgery and Breast Surgery, Asahikawa Medical University Hospital, 2-1-1-1 Midorigaoka Higashi, Asahikawa, Hokkaido 078-8510, Japan; Department of Diagnostic Pathology, Asahikawa Medical University Hospital, 2-1-1-1 Midorigaoka Higashi, Asahikawa, Hokkaido 078-8510, Japan; Department of Respiratory Medicine, Nayoro City General Hospital, 1, West 7, South 8, Nayoro, Hokkaido 096-8511, Japan; Department of Thoracic Surgery and Breast Surgery, Asahikawa Medical University Hospital, 2-1-1-1 Midorigaoka Higashi, Asahikawa, Hokkaido 078-8510, Japan

**Keywords:** granulocyte colony stimulating factor, antiphospholipid antibody syndrome, lung cancer

## Abstract

No reports on granulocyte colony-stimulating factor-producing lung cancer associated with antiphospholipid antibody syndrome. A 73-year-old man was referred to our department to undergo surgery for lung cancer in the right upper lobe. His examination results suggested that his condition was caused by an elevated white blood cell count and an increased inflammatory response due to granulocyte colony-stimulating factor production. The presence of antiphospholipid antibody syndrome was suspected, and the decrease in coagulation factors was considered to be inhibited by the lupus anticoagulant. Perioperatively, the patient was treated with heparin and steroids, and a thoracoscopically assisted right upper lobectomy was performed. Postoperatively, histopathological examination revealed pleomorphic carcinoma, and the patient tested negative for anticardiolipin IgG antibodies. In lung cancer patients with elevated white blood cell counts, fever, and an inflammatory response, granulocyte colony-stimulating factor-producing lung cancer is an important differential diagnosis. Additionally, when coagulation abnormalities are observed preoperatively, a thorough examination is necessary to prepare for perioperative management.

## Introduction

Granulocyte colony stimulating factor (G-CSF) is a cytokine involved in granulocyte proliferation, and tumor cells reportedly produce G-CSF in lung cancer with leukocytosis as G-CSF-producing lung cancer [1]. Antiphospholipid antibody syndrome (APS) is a disease caused by autoantibodies called antiphospholipid antibodies, which lead to thrombosis of the arteries and veins [2].

## Case presentation

**Figure 1:**
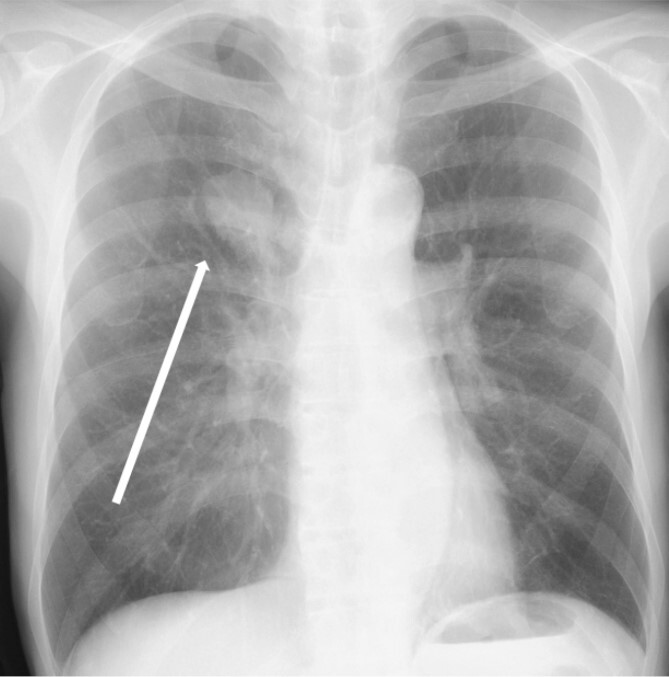
Chest X-ray findings (frontal view). Mass shadow in the upper lobe of the right lung.

**Figure 2:**
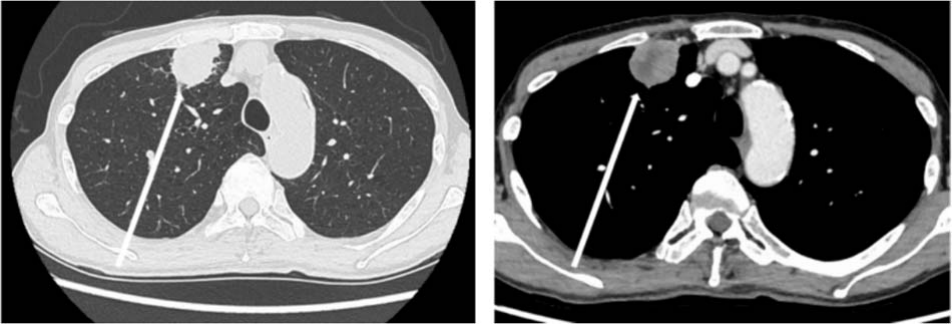
Chest computed tomography (CT) findings. An irregular mass 43 × 33 × 28 mm in size was found in the upper lobe of the right lung, which was suspected to be primary lung cancer.

**Figure 3:**
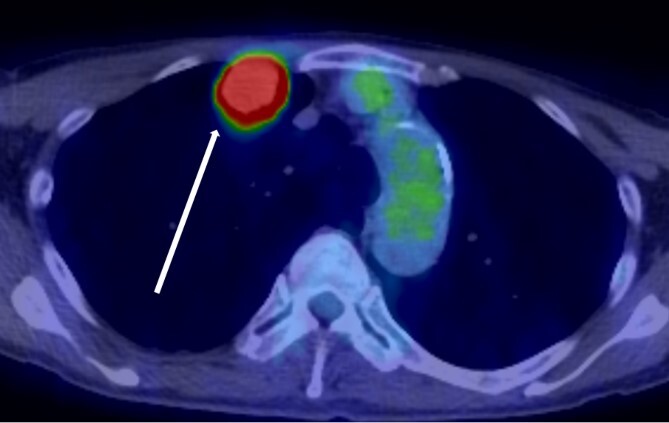
^18^F-fluorodeoxyglucose positron emission tomography findings. Abnormal accumulation of Max SUV 28.4 was found in a pointed lesion in the right upper lobe of the lung, which was considered to be a primary lung cancer site.

The patient was a 73-year-old man. He had a history of arteriosclerosis obliterans in the lower extremities, transient atrial fibrillation, and was consuming two antiplatelet agents. The patient had a fever within the range of 38°C. Blood tests showed an elevated white blood cell (WBC) count of 31.6 × 10^3^/μl (neutrophil, 90.2%), CRP of 9.67 mg/dl, and APTT of 125.4 s. Chest radiography revealed an abnormal shadow in the upper lobe of the right lung (Fig. 1). Contrast-enhanced chest CT revealed an irregularly shaped mass, 43 × 33 × 28 mm in size, in the upper lobe of the right lung (Fig. 2). ^18^F-fluorodeoxyglucose-positron emission tomography showed abnormal accumulation with a maximum standardized uptake value of 28.4 in a pointed lesion in the upper lobe of the right lung (Fig. 3). A bronchoscopic biopsy was performed, and the patient was diagnosed with non-small cell carcinoma.

Additional examinations, including bone marrow examinations and cross-mixing tests, were performed in collaboration with the hematology department. Bone marrow examination revealed no obvious abnormalities. Cross-mixing test (APTT) showed no linear correction, and lupus anticoagulant was considered a possible cause. Subsequently, blood samples were collected and analyzed for lupus anticoagulant, showing the following results: lupus anticoagulant, 2.2; anti-cardiolipin IgG antibody, 10.8 U/ml; anti-beta2GPIIgG antibody, 71.9 U/ml; G-CSF, 164 pg/ml. Prednisone 10 mg infusion and antipyretic analgesics were initiated, and the WBC count decreased. One week before surgery, the two antiplatelet agents were discontinued, and heparin replacement was initiated at a dose of 10 000 U/day. Heparin was discontinued on the day of surgery, and 500 mg hydrocortisone sodium phosphate and 10 mg prednisone were administered intravenously.

**Figure 4:**
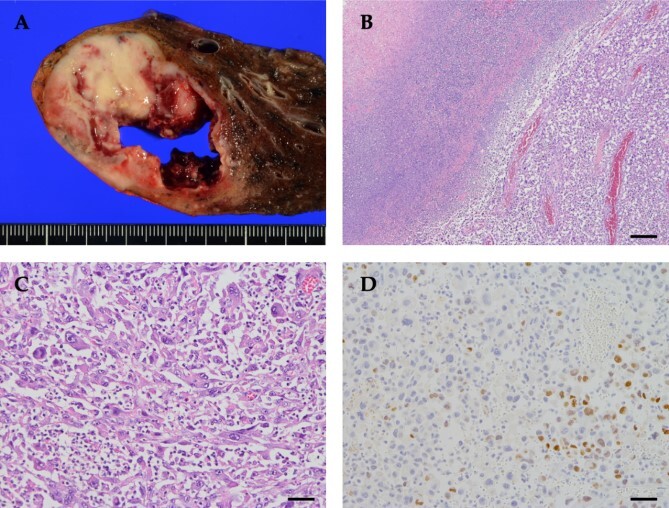
Pathological findings of the tumor. (A) Grossly, a tan to yellowish tumor with necrosis was observed. (B) The tumor cells were less cohesive, accompanied by abscess and necrosis (top left). (C) The tumor was composed of spindle-shaped cells, polygonal cells with bizarre nuclei, and multinucleated giant cells, with prominent neutrophil infiltration between tumor cells. (D) Tumor cells were focally positive for TTF-1. Scale bar: (B) 250 μm, (C and D) 50 μm.

Routine upper lobectomy was performed. A 5.5 × 5.3 × 4.0 cm tumor was observed on the split surface bordering the pleura. Histopathological examination revealed atypical cells with irregularly shaped nuclei of unequal size. Large bizarre nuclei, multinucleated cells, and spindle-shaped cells occupied ~75% of the tumor. Immunohistochemistry analysis showed that the tumor was partially TTF-1-positive and p40-negative, with a poorly differentiated adenocarcinoma component (Fig. 4). The patient was finally diagnosed with pleomorphic carcinoma with an adenocarcinoma component, pT3N0(0/17)M0, pStage IIB. G-CSF immunohistochemistry was not performed because it was not available at our hospital.

Postoperatively, heparin replacement was left unterminated, and antiplatelet agents were resumed the next day. Blood tests showed a WBC count of 13.6 × 10^3^/μl (neutrophil, 74.2%) and a CRP of 2.12 mg/dl. With a lupus anticoagulant ratio of 2.46, his lupus anticoagulant remained positive; however, his anti-cardiolipin IgG antibody level was 11.0 U/ml, and his anti-beta2GPIIgG antibody level was 1.1 U/ml, showing negative results.

## Discussion

G-CSF-producing lung cancer was an important differential diagnosis in a lung cancer patient with a markedly elevated WBC count, fever, and inflammatory responses [1, 3]. The proposed diagnosis criteria for G-CSF-producing lung cancer are as follows: marked leukocytosis, mainly of mature neutrophils, without any cause; high serum G-CSF levels; decreased leukocyte counts and G-CSF levels due to tumor resection or treatment; and evidence of G-CSF production in the tumor [1]. G-CSF-producing lung cancer has a poor prognosis, sometimes <1 year, and the most common histologic type is large cell carcinoma, followed by adenocarcinoma and squamous cell carcinoma [1, 4]. Our patient had pleomorphic carcinoma, which is a relatively rare histological type.

Recent studies have reported cases of positive anaplastic lymphoma kinase gene rearrangement [5] and resistance to PD-1/PD-ligand 1 inhibitors in G-CSF-producing spindle cell carcinoma [6]. In addition, G-CSF produced by NR-S1M cells in squamous cell carcinoma promotes tumor progression in mice through its bifunctional effects on neutrophil recruitment and tumor cell growth [7]. Therefore, reports on G-CSF-producing lung cancers are expected to increase in the field of lung cancer, where treatments, including gene therapy, are rapidly evolving. At the same time, the patient also had APS, and it is very interesting that the APS status was lifted on blood tests after resection of the G-CSF-producing lung cancer, although the relation to APS is unknown.

When coagulation abnormalities are detected preoperatively, a thorough examination is necessary to prepare for perioperative management. The patient in this case had prolonged coagulation; however, he had APS with positive anticardiolipin antibodies and was thrombophilic rather than hemorrhagic. Deep vein thrombosis and cerebrovascular disease are common symptoms of APS, and anticardiolipin antibodies and loop anticoagulants are commonly used for diagnosis [2]. Elevated APTT reflects the presence of lupus anticoagulant, which suggests that the patient is at risk of thrombosis rather than bleeding [8]. A prolonged PT indicates that the patient may have antibodies against prothrombin (factor II); thus, patients with APS are at high risk of thrombosis and require caution during the perioperative period. Although there is no definitive data on the perioperative management of APS, the general rule of thumb is to use precise anticoagulation therapy and to ambulate as early as possible after surgery [2]. Bleeding reportedly occurs more frequently than thrombosis in patients with APS and is associated with suboptimal perioperative anticoagulation management and a preoperative PT-INR > 1.5 [9]. Antithrombotic agents and glucocorticoids may also be recommended for patients with APS [10]. In this case, in addition to heparin replacement, steroids were administered during the perioperative period, and the patient was carefully managed in collaboration with the hematology department, resulting in no thrombosis and good intraoperative bleeding control.

In conclusion, G-CSF-producing lung cancer was an important differential diagnosis in a lung cancer patient with a markedly elevated WBC count, fever, and inflammatory responses. Additionally, when coagulation abnormalities are detected preoperatively, a thorough examination is necessary to prepare for perioperative management. As no cases of G-CSF-producing lung cancer complicated by APS have been reported, we believe that our report on the perioperative course of this case has a significant value.

## Data Availability

Data sharing is not applicable to this article because no datasets were generated or analyzed in the current study.
